# Effect of Sodium Hexametaphosphate and Trisodium Phosphate on Dispersion of Polycarboxylate Superplasticizer

**DOI:** 10.3390/ma12244190

**Published:** 2019-12-13

**Authors:** Yan Zhang, Huaqing Liu, Jialong Liu, Ruiming Tong

**Affiliations:** China Electric Power Research Institute, Beijing 100192, China; lhq@epri.sgcc.com.cn (H.L.); liujialong@epri.sgcc.com.cn (J.L.); tongrm@epri.sgcc.com.cn (R.T.)

**Keywords:** phosphate, precipitation, adsorption, combination, adsorption layer, interface

## Abstract

Enhancement in dispersion of polycarboxylate superplasticizer (PCE) could be obtained by incorporating retarders in normal concrete. The generally believed reason was that the consumption of free water and polymer at the beginning was reduced by retarding cement hydration. This theory could not convincingly explain why sodium hexametaphosphate (SHMP) was able to promote the dispersion capacity of PCE, while trisodium phosphate (TSP) could not, despite that both TSP and SHMP could obviously retard the cement hydration. The adsorption behavior of PCE and phosphate was investigated and the mechanism was analyzed in order to gain deeper understanding. The results showed that TSP and SHMP delayed the cement hydration, impeded adsorption process of PCE, and increased thickness of adsorption layer. It was interesting that TSP reduced the dispersion, but SHMP enhanced. The reason for this contradiction was due to the difference in composition of adsorption layer. In the PCE-TSP system, this layer was composed of the precipitates (formed by TSP and Ca^2+^) and the invalided PCE (caused by these precipitates in the immediate vicinity of the cement grains); the invalided PCE was due to the decrease of PCE dispersion. In the PCE-SHMP system, “Inner-phosphate (multi-layers) + Outer-PCE (single layer)” structure was formed to make the PCE work more effective, hence enhancing the dispersion. These results were expected to be useful for the design of highly efficient dispersants.

## 1. Introduction

In recent years, high fluidity cement-based materials have been widely used in self-compacting concrete, pumping concrete, indoor floor, and grout, mainly because the superior workability could obtain the highly efficient construction process [1,2,3,4,5,6]. However, how to efficiently disperse the cement particles and obtain excellent fluidity performance without any bleeding and segregation is essential for this kind of material [7,8], and it also ensured the durability of concrete [9,10,11,12]. Generally, high fluidity can be easily achieved by incorporation of the comb-type copolymers, known as polycarboxylate superplasticizer (PCE) system [13,14,15,16], and this system consists of copolymers and retarders, rather than a single-component system [17,18,19]. In spite of being added as a supplementary material, retarders can significantly enhance the dispersion capacity of the superplasticizer system [20,21], and, in the real engineering practice, this has been widely employed. The generally believed reason is because the consumed free water and superplasticizer was reduced, as a result of the delayed hydration of cement minerals [22,23,24,25]. However, it has been proven that polyethylene oxide (PEO) side chains offer the main dispersion force (i.e., steric hindrance). It is noted that the polymer cannot exert dispersion unless it can adsorb on the surface of cement grains (i.e., S-PCE), and more S-PCE would provide higher dispersion capacity [26,27,28,29,30]. Based on this, the retarder might hinder the adsorption process of PCE and it was referred to as competitive adsorption [31,32,33,34,35], thereby reducing the dispersion. In addition, the thickness of the adsorption layer should also be accepted as a factor influencing the dispersion of PCE [36,37]. In spite of the decline in adsorption amount of PCE in the presence of retarder, as reported in previous study, the adsorption layer was obviously thickened to enhance the dispersion [37]. On the basis of discussion above, the conclusion could be made that the dispersion capacity of the PCE-retarder system should be associated with the retarding effect of retarder, adsorption behavior of PCE, and the thickness of the adsorption layer.

Poly-phosphates, such as sodium tripolyphosphate and sodium hexametaphosphate (SHMP), have been widely used to promote the dispersion ability and dispersion retention ability of the superplasticizer system in real engineering practice. However, mono-phosphates, such as trisodium phosphate (TSP) and sodium hydrogen phosphate, cannot play the same roles as poly-phosphate in commercially available PCE products. If the retardation was the main reason for the enhancement, poly-phosphate and mono-phosphate would both have a similar effect on dispersion of PCE system, because both TPS and SHMP could retard the cement hydration [38]. Therefore, the reason why the poly-phosphate could effectively enhance the dispersion, whilst the mono-phosphate failed cannot be simply explained by the retarding effect. One possible explanation might be attributed to the difference in the molecular structure of the phosphates, which could result in different performance at the water/solid interface during the very early-stage hydration [39,40,41,42], and this would further influence the adsorption layer [43,44,45]. As a consequence, the difference in PCE adsorption behavior could be expected in the presence of different phosphates.

In this study, the dispersion mechanism of the PCE-phosphate system was investigated. The rheology of cement paste was tested to evaluate the dispersion capacity of PCE-phosphate system. Adsorption behavior, including adsorption amount and thickness of adsorption layer, was analyzed to evaluate the water/solid interface performance of cement particles. The adsorption model was also proposed and the dispersion mechanism behind was revealed. Such results were expected to provide guidance regarding how to promote the dispersion capacity of PCE system in the future.

## 2. Experimental

### 2.1. Materials

#### 2.1.1. Cement

An ordinary Portland cement (P. O. 42.5, supplied by Yadong Co., Ltd., Wuhan, China), in accordance with the requirements of GB 175-2007 Chinese standard, was used, and Table 1 shows the chemical compositions.

#### 2.1.2. Polycarboxylate Superplasticizer

**Figure 1:**
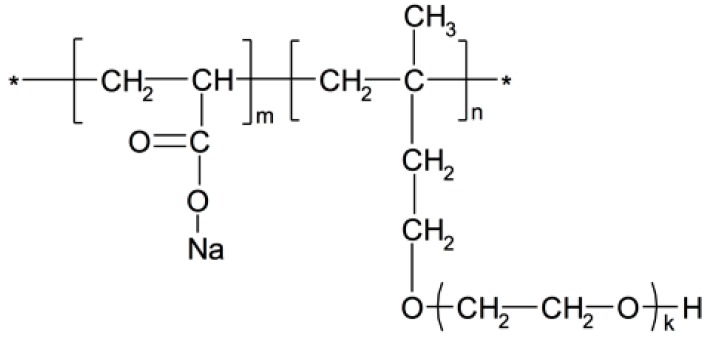
Molecular structure of polycarboxylate superplasticizer (PCE) (m:n = 3.5:1; k = 48–52).

Table 2 and Figure 1 show the basic performance and the molecular structure of PCE (the information was offered by HuanXuan Co., Ltd., Wuhan, China). From the figure, it was found that the mole ratio of carboxyl groups and long side chain was 3.5:1. Additionally, the polymerization degree of ethylene oxide was 48–52.

#### 2.1.3. Phosphates

**Figure 2:**
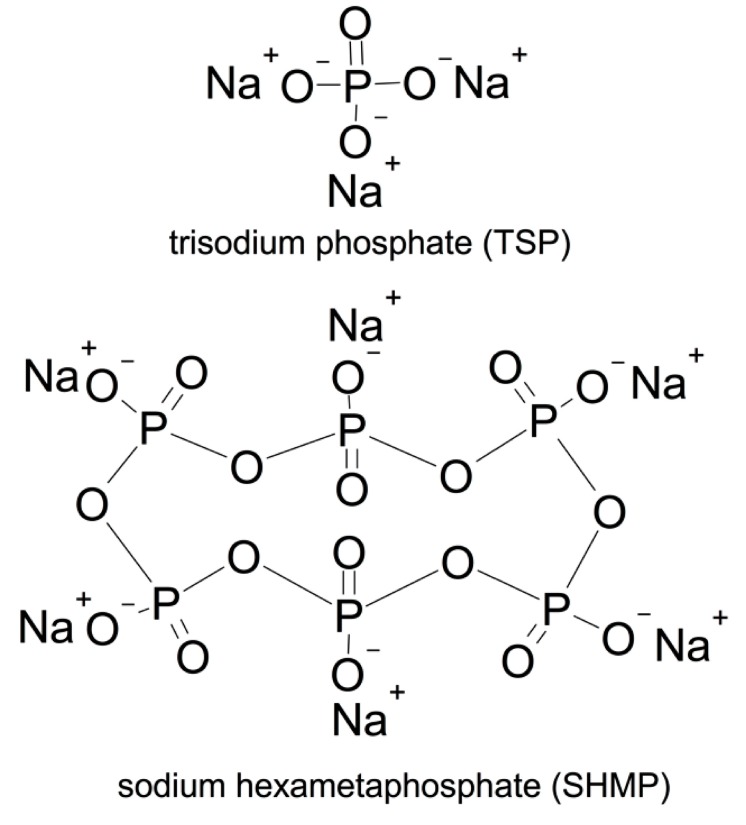
Molecular structure of phosphates.

The reagent grade phosphates, including TSP (Na_3_PO_4_·12H_2_O, the crystal water was considered in the experiment) and SHMP (Na_6_P_6_O_18_, supplied by Sinopharm Chemical reagent Co., Ltd., Shanghai, China), were used. Figure 2 shows the molecular structures. From the figure, it was clearly observed that TSP is categorized as a mono-phosphate, while SHMP is a poly-phosphate.

### 2.2. Test Methods

#### 2.2.1. Rheology

PCE and phosphate were added in water in advance, and the solution was then mixed with cement. Cement pastes in the presence of PCE-phosphate were prepared with 0.29 water/cement weight ratio (water: 87 g, cement: 300 g), and 0.44 water/cement weight ratio was used in pastes with phosphate, in accordance with the requirements of Chinese standard GB 8076-2008.

The rheology was evaluated by Rotor rheometer (R/S-SST, rotor: CC45, made by Brookfield, WI, USA). To reach a reference, the paste was firstly pre-sheared at a shear rate of 120 s^−1^ for 30 s, and a shear rate was then directly applied from 0–100 s^−1^ within 120 s, followed by a shear rate from 100–0 s^−1^ within 60 s. Rheo 2000 V2.8 software (supplied by Brookfield, Middleboro, MA, USA) was used to process the data, and each flow curve was fitted with Bingham model and the value of yield stress and plastic viscosity was calculated.

#### 2.2.2. Adsorption Amount

**Figure 3:**
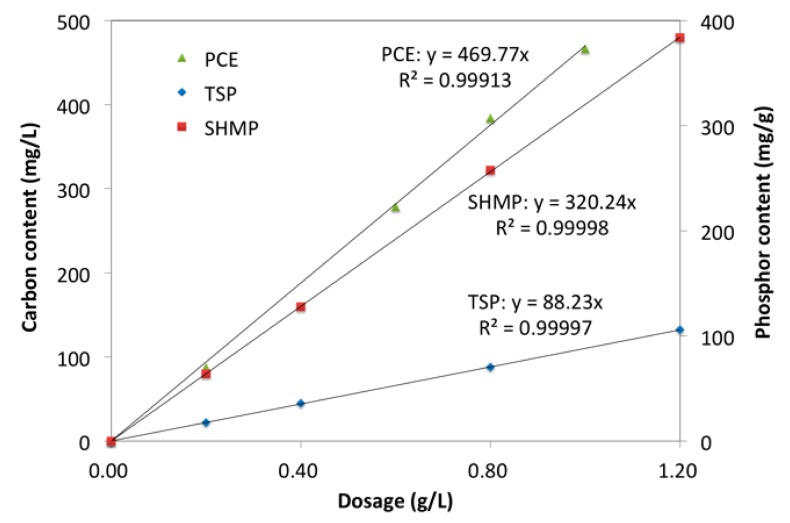
Relationship between concentration and tested results.

Phosphate solutions (0.20 g/L, 0.40 g/L, 0.80 g/L, and 1.20 g/L) were prepared in advance and the phosphor content of the solution was examined by inductive coupled plasma emission spectrometer (ICP, Optima 4300 DV, made by Perkin Elmer Ltd., Wellesley, MA, USA). With the same step, total organic carbon analyzer examined carbon content (TOC, Liquid TOC II, made by Elementar, Germany). Figure 3 presents the results. Based on these results, the concentration of phosphate or PCE in solution was calculated with the phosphor content and carbon content tested by ICP and TOC, respectively.

The solutions (20.0 g, components are shown in Table 3) were prepared in advance. These solutions and cement (1.0 g) were mixed together and stirred for 5 min., respectively. The suspension was separated by centrifugation at 3000 r/min for 4 min. The solid was dried in a vacuum at 25 °C and then prepared for the measurement of X-ray photoelectron spectrometer (XPS), and the upper supernatant was prepared for the measurements of TOC and ICP. Three times text was done in each measurement, and the average was the results. Based on those results shown in Figure 3, the concentration of PCE and phosphate in the upper supernatant (i.e., residual concentration) was obtained from the results of TOC and ICP. The adsorption amount (mg/g-cement) and adsorption ratio was calculated, as follows:

Adsorption amount = V (C_0_-C)/m

Adsorption ratio = (C_0_-C)/C_0_

where, C_0_ represented the concentration of PCE or phosphates before adsorption, g/L; C denoted the concentration after adsorption, g/L; V was the volume of the solution, mL; m was the mass of the cement, g.

#### 2.2.3. Adsorption Layer

The surficial silicon and calcium were tested by X-ray photoelectron spectrometer (XPS, XSAM 800, made by KRATOS, England, UK); aluminum was used as an anode target (*hν* = 1486.6 eV); energy resolution was 0.100 eV. XPSPEAK 4.1 was used to process the data of XPS. The change of the binding energy of Ca2p indicated that the chemical bond of calcium on cement surface was altered, which provided evidence to prove the formation of the new type calcium-based compound. Furthermore, the adsorption layer could cover the silicon layer and reduce the intensity of the photoelectron. The relationship between the thickness of adsorption layer and intensity of photoelectron was shown, as follows:

I(b) = I_0_exp[−b/λ(E_k_)]

λ(E_k_) = 49E_k_^2^ + 0.11(E_k_)^1/2^

E_k_ = *hν* − E_b_

where,

I_0_ denoted the initial photoelectron intensity;
I(b) represented the photoelectron intensity after the photoelectron went through the adsorption layer;
meant the thickness of adsorption layer, nm;
E_k_ was the photoelectron kinetic energy after the photoelectron went through the adsorption layer, eV;
E_b_ was the electron binding energy, eV.

Based on this, the thickness of adsorption layer could be calculated from the XPS data of silicon [37].

#### 2.2.4. Hydration Heat

Phosphates (0.20 wt% of cement) were added into water in advance, respectively; and then, the paste was prepared with a water/cement weight ratio of 0.35:1. Hydration heat of cement paste was obtained by micro calorimeter.

#### 2.2.5. Phase Analysis

Cement pastes with phosphates (0.20 wt% of cement) were prepared with a 0.35 water/cement weight ratio. After being cured for one day under the room temperature (20 °C) and relative humidity (RH) higher than 90%, they were broken into small pieces and then immersed in anhydrous ethanol to stop hydration. After that, the samples were dried under vacuum and ground to pass 63 um sieve. The powders are used for the measurement of X-ray Diffractometer (XRD, D/Max-RB, made by Rigaku, Japan) with Cu (Kα) radiation and a current of (40 mA, 40 kV), at a speed of 4°/min. and a step of 0.02° within the range of 5 to 70°.

#### 2.2.6. Conductivity Measurement

The solution of calcium hydroxide (CH, 0.10 g/L), PCE (0.1 g/L, 10.0 g/L), SHMP (1.0 g/L), and PCE-SHMP (PCE: 10.0 g/L, SHMP: 1.0 g/L) were prepared in advance. The conductivity of deionized water, PCE solution, SHMP solution, and PCE-SHMP solution with the increasing dosage of CH was examined with an electrical conductivity meter. The conductivity of deionized water and SHMP-CH with the increasing dosage of PCE was tested with the electrical conductivity meter. The reference was the deionized water. The different tendencies from the reference were able to illustrate the reaction happening in liquid phase.

## 3. Results and Discussions

### 3.1. Rheology of Cement Paste Plasticized by PCE-Phosphate

**Figure 4:**
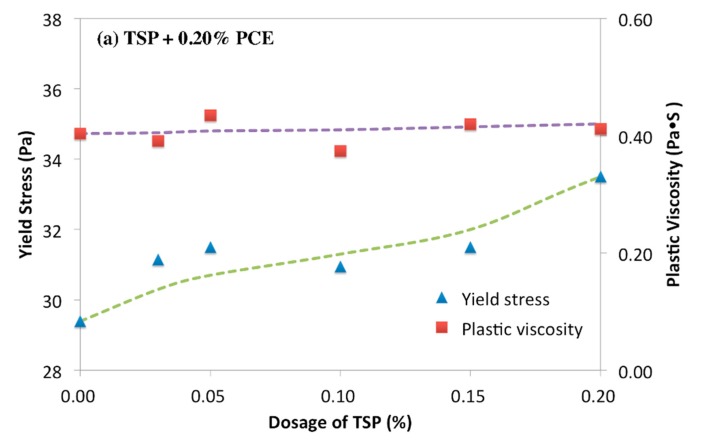

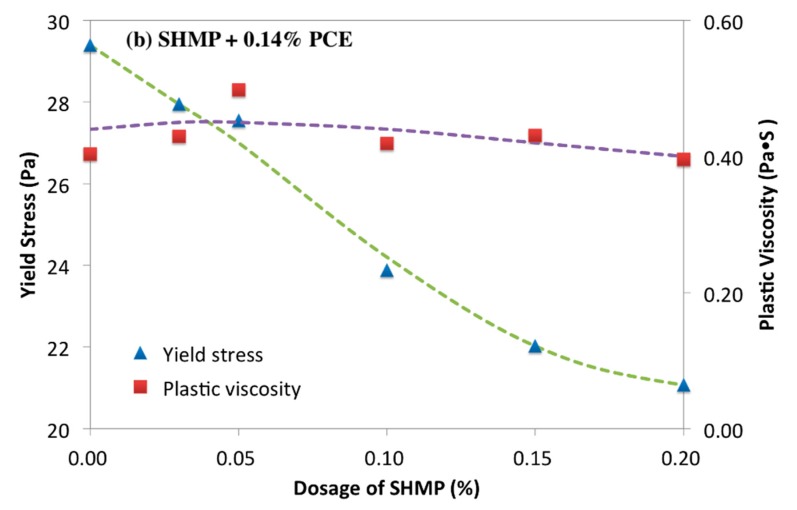
Rheology of cement paste with PCE-trisodium phosphate/sodium hexametaphosphate, (**a**) PCE-TSP; (**b**) PCE-SHMP.

Figure 4 shows the yield stress and viscosity of cement paste in the presence of PCE-phosphate. As shown in Figure 4a, it was found that the yield stress of the paste with PCE-TSP was increased with the increasing dosage of TSP, while the plastic viscosity was slightly increased. This result illustrated that TSP declined the dispersion of PCE. It was obviously different from what it was expected that TSP should enhance the dispersion due to the retarding effect. Furthermore, in cement paste with the addition of PCE-SHMP, as shown in Figure 4b, the decline in yield stress was observed with the increasing dosage of SHMP, and this result indicated that SHMP could enhance the dispersion of PCE, which was opposite to the result of TSP. The plastic viscosity was firstly increasing and then reduced, and this phenomenon was revealed, as follows: on the one hand, due to the plasticizing effect, the agglomerated particles were dispersed, and finer particles in suspension was increased to promote the plastic viscosity [46,47]. On the other hand, the amount of free water in system would be increased to decrease the plastic viscosity due to the retarding effect. Accordingly, the predominated aspect should determine plastic viscosity. The increased stage of plastic viscosity indicated that the dispersion effect was predominated when the dosage of SHMP less than 0.10%, while the decrease indicated that the retarding effect on hydration predominated when the added dosage was more than 0.10%.

**Figure 5:**
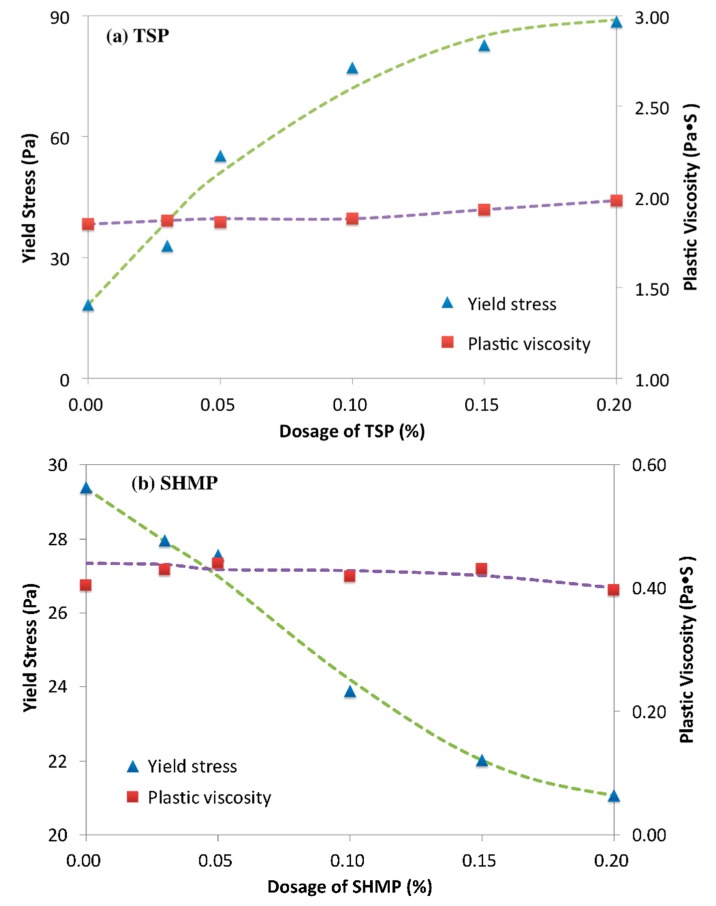
Yield stress and plastic viscosity of cement paste with TSP/SHMP, (**a**) TSP; (**b**) SHMP.

The effect of single phosphate system on the rheology of cement paste was also considered, and the results are shown, as follows:

From Figure 5a, it was found that the yield stress was slightly increased from 29.4 Pa to 33.5 Pa by adding TSP, while the plastic viscosity was almost kept constant. As shown in Figure 5b, the yield stress of the cement paste was declined by SHMP; this illustrated the plasticizing effect of SHMP, and the possible reason was the reduced consumption amount of free water originating from the delay of cement hydration and the increased zeta potential resulting from adsorption. Furthermore, the plastic viscosity was slightly reduced, and this was attributed to the delayed formation of hydration products and the reduced yield stress.

On the basis of the discussion above, it was concluded that, in cement paste, SHMP showed the obvious plasticizing effect, but TSP not, and in PCE-phosphate system, dispersion of PCE was enhanced by SHMP, but reduced by TSP. This agreed with the practical experience. Conventionally, retarder could delay the cement hydration and hydrate formation, which would save free water to reduced yield stress of cement paste. This could explain the reduced yield stress by SHMP, but it could not reveal the reason for the reduced yield stress by TSP. In the PCE-phosphate system, the positive effect of SHMP on rheological performance could be explained from the retarding effect of SHMP, while that for TSP seemed contradictory.

### 3.2. Retarding Effect of Phosphates on Cement Paste

**Figure 6:**
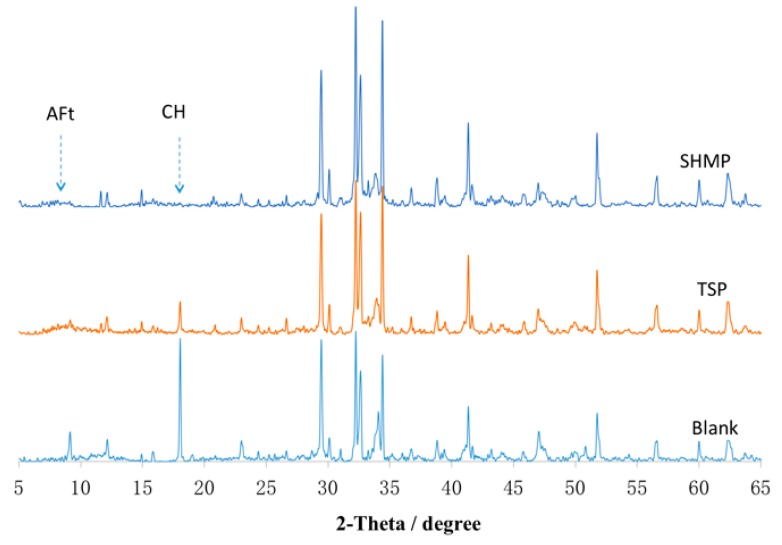
X-ray Diffractometer (XRD) pattern of cement paste with phosphate hydrating for 1 d.

**Figure 7:**
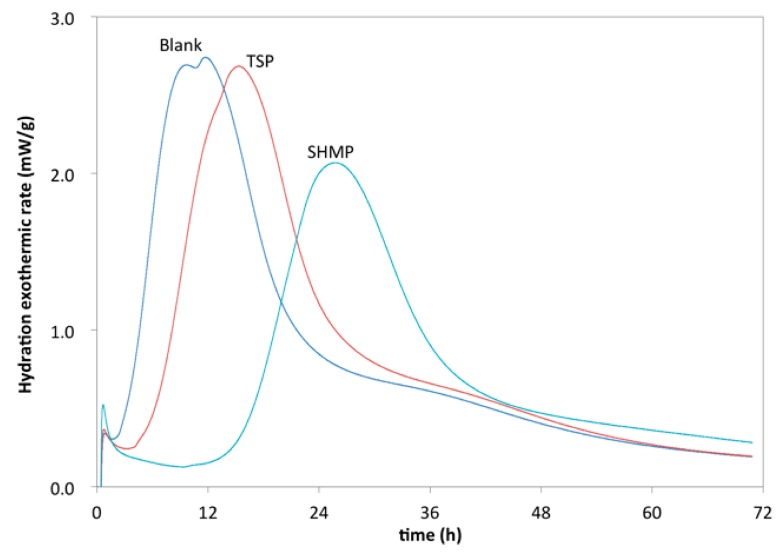
Hydration heat of the cement paste with phosphate.

The noticeable difference in the retarding effect of the phosphates was clearly found from XRD and hydration heat. CH (i.e., calcium hydroxide) and AFt (i.e., ettringite) are considered as typical hydration products of Portland cement, and generally, the degree of hydration of the cement can be inferred from the amount of these two kinds of hydration products [48,49,50,51], which can be reflected in the characteristic peak in XRD. As shown in Figure 6, a much lower characteristic peak of CH and AFt than that of the reference was observed, indicating that both TSP and SHMP delay the hydration of the cement paste in the early age and SHMP showed stronger retarding effect than TSP. The same result was also found in the hydration heat, as shown in Figure 7. TSP and SHMP both delayed heat peak, but SHMP showed a stronger effect than TSP. This result also indicated that TSP and SHMP could both delay the cement hydration, and SHMP showed a stronger retarding effect than TSP; these results would be most likely related to the adsorption of chemicals [52,53].

From the discussion above, SHMP and TSP could both be deemed as retarder in the cement hydration process, and SHMP has stronger retarding effect than TSP. Based on conventional theory, both TSP and SHMP should enhance the dispersion of PCE, rather than that TSP reduced the dispersion. The enhancement could be explained reasonably, while the decline could not. As a consequence, the mechanism behind could not be only explained from retarding effect.

### 3.3. Adsorption Amount

**Figure 8:**
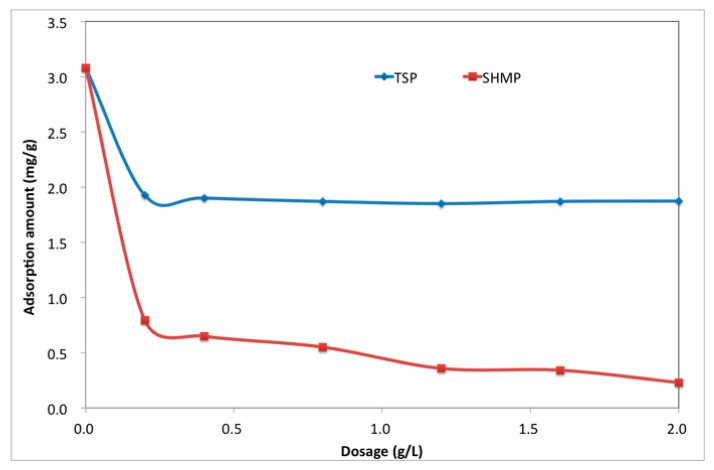
Effect of TSP and SHMP on adsorption of PCE (PCE: 1.0 g/L).

The adsorption amount of PCE has been reported to determine the dispersion ability of PCE [26]. In the presence of retarder, competitive adsorption would happen between PCE and retarder; it meant that the adsorption process of PCE would be hindered by the addition of phosphate, which resulted in a decline in the dispersion [20,31,54]. The adsorption amount of PCE in the presence of TSP and SHMP was tested in order to confirm the competitive adsorption, and Figure 8 shows the results. It was found that TSP and SHMP could both significantly decrease the adsorption amount of PCE, and the ability of SHMP to hinder PCE from adsorption was much stronger than that of TSP. This result illustrated that the dispersion ability of PCE should be reduced by the presence of phosphates. Based on this, the decrease in fluidity and increase in yield stress caused by PCE-TSP was reasonably explained. However, this could not reveal the reason for the opposite phenomenon with PCE-SHMP. As a consequence, this mechanism could not be simply explained by competitive adsorption as well.

**Figure 9:**
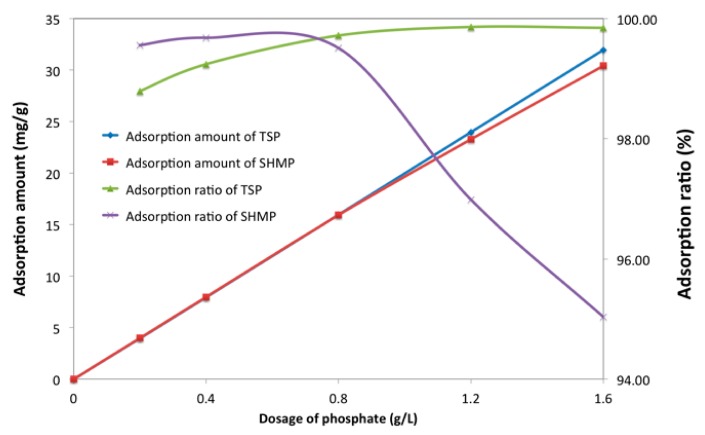
Adsorption of phosphate in cement suspension.

The adsorption behavior of phosphate in cement suspension was investigated to further discuss the competitive adsorption, and the results are shown in Figure 9.

TSP and SHMP were both able to adsorb on the surface of the cement grains, and the adsorption ratio reached more than 95.0%, which meant that the vast majority of the phosphate could adsorb onto the surface of the cement particles. In liquid phase, phosphates were quickly ionized and released the anions of [PO_4_]^3−^ or [P_6_O_18_]^6−^, with negative charge. On the one hand, because of electrostatic attraction resulting from the positive zeta potential of cement particles at the very beginning [55], the ionized phosphate could quickly adsorb onto the cement surface. On the other hand, the released Ca^2+^ into solution that was caused by cement hydration could be precipitated with [PO_4_]^3−^ or be combined with [P_6_O_18_]^6-^, which results in adsorption. In other words, the adsorption of TSP resulted from the precipitation with Ca^2+^ in the immediate vicinity of cement particles, and adsorption of SHMP was due to the combination with surficial Ca-O or Ca^2+^. In that case, these phosphates occupied the adsorption point and, therefore, PCE could not adsorb on these points, which was inferred to be responsible for the reduced adsorption amount of PCE by adding phosphate.

**Figure 10:**
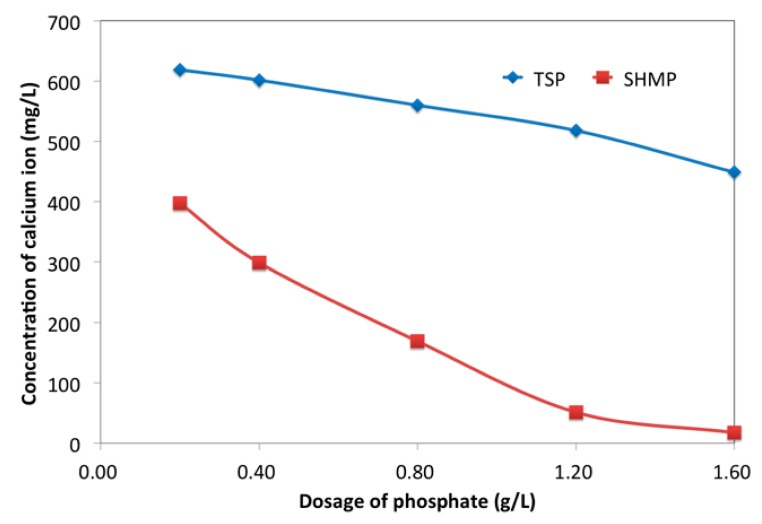
Effect of phosphates on Ca^2+^ in pore solution.

In addition, the adsorption of phosphate would hinder the release of Ca^2+^ into the liquid phase. This hindrance was directly proved by Ca^2+^ concentration in pore solution, as displayed in Figure 10. TSP and SHMP both reduced the concentration of Ca^2+^ in pore solution. Combined with the analysis of adsorption, the reduced concentration of Ca^2+^ indicated that the formation a calcium-based phosphate layer. It was this layer that resulted in the retarding effect of phosphate. Furthermore, this layer hindered the Ca^2+^ from being released into solution, thereby delaying the formation of hydration products; one the other hand, it isolated water from cement particles to hinder the cement hydration.

### 3.4. Thickness of Adsorption Layer

The adsorption layer was reported to affect the dispersion ability of PCE and, generally, the thicker adsorption layer was able to provide a stronger dispersing ability [54,56]. Thickness of adsorption layer in the presence of PCE (0.40 g/L) and phosphate (0.40 g/L) was calculated from the XPS data of Si2p, and Table 4 shows the results. From the table, the strongest intensity of photoelectron was observed in the blank sample, and the decreased intensity of photoelectron in the presence of PCE or PCE-phosphate system indicated the existence of adsorption layer on the surface of the cement particles.

As shown in Table 4, TSP and SHMP in PCE system both increased the thickness of the adsorption layer, which might promote the dispersion capacity of the PCE-phosphate system. This could explain the increased dispersion ability of PCE-SHMP, but it was unclear why dispersing ability of PCE could not be promoted by adding TSP. As a consequence, the mechanism could not be simply explained by the thickness of adsorption layer.

### 3.5. Composition of Adsorption Layer

**Figure 11:**
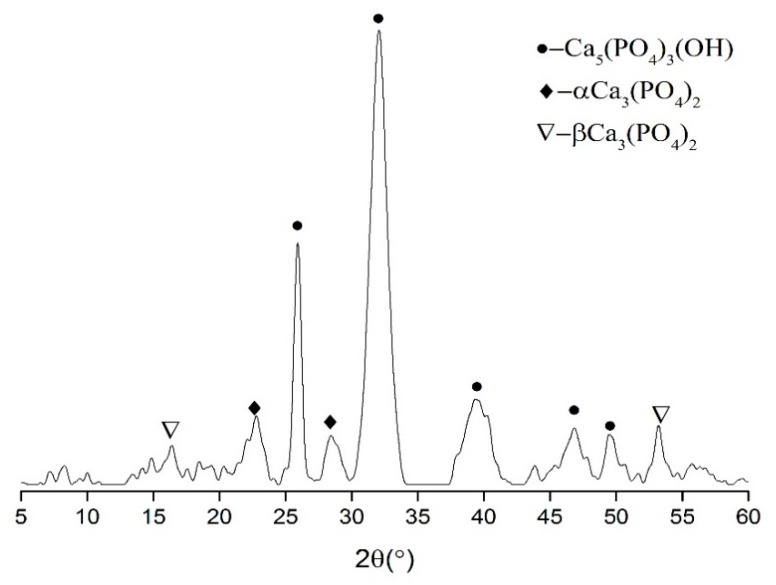
XRD pattern of the precipitate.

The composition of adsorption layer was further discussed to reveal the mechanism:

In the PCE system with TSP, the white precipitates were observed in the solution almost at the same time with the addition of calcium hydroxide solution (CH, 0.10 g/L). The solid in suspension was filtered and dried at about 25 °C in order to confirm the chemical composition of the precipitate; then, these powders were tested by XRD. As shown in Figure 11, it was seen that the precipitate was composed of hydroxyapatite and calcium phosphate. This result indicated that hydroxyapatite and calcium phosphate would present on the surface of the cement grains.

On the other hand, TSP could be precipitated with Ca^2+^ in solution at a very low concentration since the solubility product constant (Ksp) of the calcium phosphate (TCP, Ca_3_(PO_4_)_2_) and hydroxyapatite (AH, Ca_10_(PO_4_)_6_(OH)_2_) is 2.07 × 10^−29^ and 2.35 × 10^−59^ at the room temperature [57]. It was well known that the pH value of cement suspension could exceed 12 immediately after the cement were mixed with water [55]. The concentration of Ca^2+^ was calculated to be about 0.001–0.005 mol/L; if the concentration of [PO_4_]^3-^ was more than 1.0 × 10^−10^ mol/L, the precipitation would take place. Obviously, the precipitate could be formed almost simultaneously with the release of the Ca^2+^ [58]. This result provided enough evidence to prove that it was in the immediate vicinity of cement particles that the precipitation would happen. In that case, these precipitates most likely covered the PCE, which was adsorbed on the surface of the cement particles. Therefore, it was deduced that the adsorption layer should be composed of the mixed calcium-based phosphate and PCE polymer.

**Figure 12:**
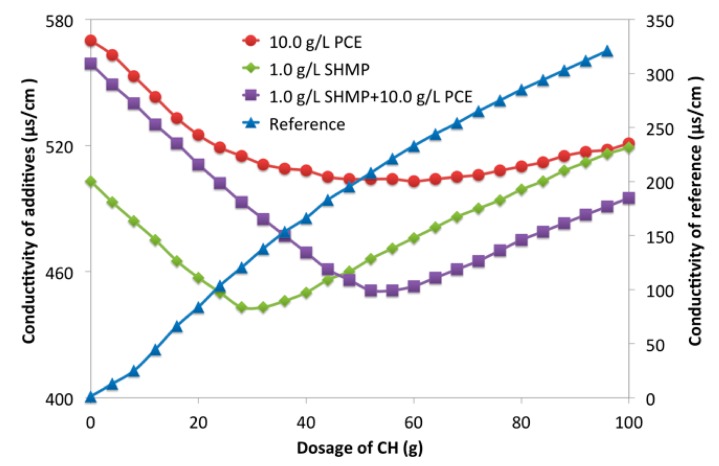
Conductivity of PCE-SHMP with CH (0.1 g/L).

**Figure 13:**
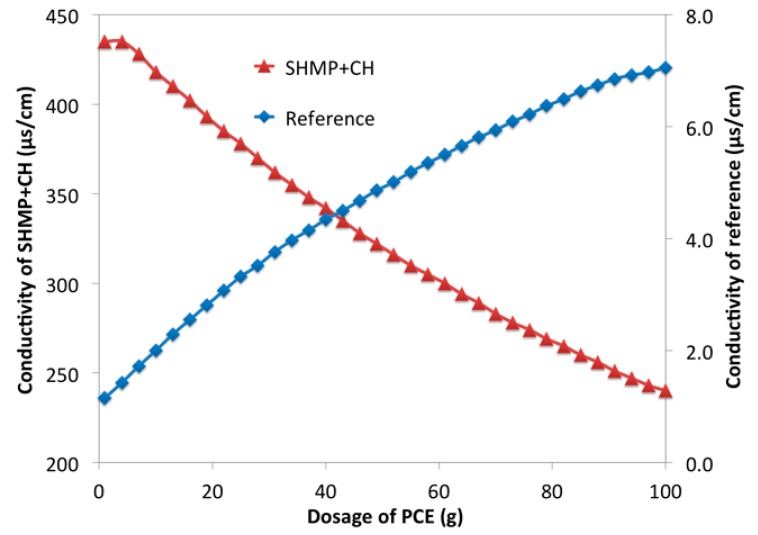
Conductivity of SHMP-CH with PCE (0.1 g/L).

In PCE-SHMP system, SHMP was combined with Ca^2+^ rather than precipitated with Ca^2+^. The released hexametaphosphate ions were combined with the surficial Ca-O or Ca^2+^ to form the complexes, thereby adsorbing onto the surface of the cement particles. They could also be combined with Ca^2+^, which presented in solution. One Ca^2+^ was combined at least two hexametaphosphate ions and then a net structure would be formed on surface of the cement particles. At the same time, Ca^2+^ was also combined with carboxyl groups of PCE, and it was possible that one PCE and one SHMP could be combined with one Ca^2+^. That was to say, one Ca^2+^ might bridge one SHMP and one PCE. The conductivity of the solution was tested with an electrical conductivity meter to further confirm the combination with Ca^2+^. In Figure 12, the conductivity of the reference (deionized water) was increased with the increasing dosage of CH (0.1 g/L), because of OH^-^ and Ca^2+^ brought by CH. For PCE, conductivity was first reduced and then increased. The decline indicated the consumption of the ion in solution, because Ca^2+^ was combined with carboxyl groups in PCE [59,60,61]. After almost all carboxyl groups were combined, the conductivity was increased. The same result was also seen in SHMP solution and PCE-SHMP solution, as shown in Figure 12. Furthermore, the combining capacity was calculated from the consuming amount of CH at the inflection point. The consumption of CH (0.1 g/L) for PCE (100 g, 10 g/L) as about 52 g, about 30 g for SHMP (100 g, 1.0 g/L), and about 56 g for PCE-SHMP (100g, 10.0 g/L PCE and 1.0 g/L SHMP). If no interaction among PCE, SHMP, and Ca^2+^, the consumption of CH (0.1 g/L) for PCE-SHMP would be 80–90 g (i.e., the summation of the consumed CH by SHMP and PCE). In fact, that consumption for PCE-SHMP was merely a little more than that for PCE, being significantly different from what was expected. This result provided strong evidence to prove that the interaction among PCE, SHMP and Ca^2+^ took place. SHMP (100 g, 1.0 g/L) was mixed with CH (30 g, 0.1 g/L) to further confirm this interaction; in this solution, the phosphate ions were almost completely combined with Ca^2+^ as SHMP-Ca (i.e., SHMP, which has been combined with Ca^2+^). Additionally, then, the conductivity of this solution with the increasing dosage of PCE solution (0.10 g/L) was measured and the change tendency was discussed. In Figure 13, PCE increased the conductivity of reference (deionized water). However, the opposite pattern was observed in SHMP-CH solution. This phenomenon could further prove the interaction between PCE and SHMP-Ca. Based on discussion above, it was concluded that PCE could be combined with SHMP-Ca to form PCE-Ca-SHMP, which meant that, in the PCE-SHMP system, the bridging Ca^2+^ between SHMP and PCE was formed. In that case, SHMP could adsorb onto the SHMP layer via the bridging Ca^2+^, and PCE could also adsorb onto the surface of the SHMP layer, because of the combination with Ca^2+^ (SHMP-Ca-PCE). Therefore, it was inferred that the adsorption layer should compose a phosphate net structure inside and a PCE layer outside.

**Figure 14:**
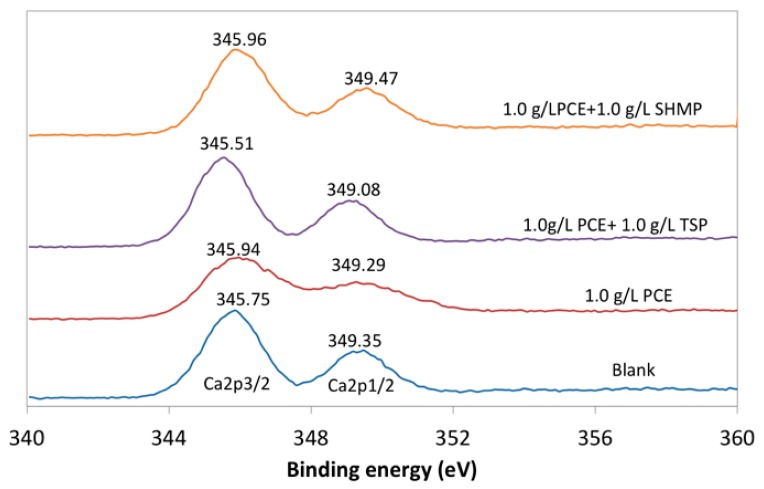
Binding energy of Ca2p on the surface of cement particle.

As mentioned above, in the PCE-phosphate system, the calcium-based phosphate layer was produced on the surface and, therefore, a new type of calcium-based compound would be formed on the surface of the cement particles, no matter whether precipitation or combination. If so, different binding energy of calcium from the blank would be expected. To confirm this, the binding energy of calcium was evaluated with XPS. As shown in Figure 14, the binding energy of Ca2p_3/2_ was increased from 345.75 eV in blank to 345.94 eV in the presence of PCE, with an increase by 0.19 eV, which indicated the formation of PCE-Ca on the surface of the cement grains. PCE-TSP reduced the binding energy of Ca2p_3/2_ by 0.43 eV, with a decrease by 0.21 eV in binding energy of Ca2p_1/2_. when compared with PCE. This result demonstrated that the calcium-based phosphate was formed on the surface of the cement particles. For PCE-SHMP, the peak shift of binding energy of Ca2p_1/2_ was 0.18 eV in comparison with that of PCE, which was caused by the formation of the complex, namely SHMP-Ca. As a consequence, the results of binding energy of Ca2p provided further evidence to confirm the precipitation or combination of phosphate with Ca^2+^ on the cement surface.

### 3.6. Dispersion Model

On the basis of discussion mentioned above, the enhanced dispersion capacity of PCE by SHMP and the reduced dispersion ability resulting from TSP could not be convincingly explained by the retarding effect, competitive adsorption, or thickness of adsorption layers. However, based analysis of composition of adsorption layer, the dispersion models were proposed to reveal the mechanism behind as follow:

Steric hindrance, provided by PEO chain, has been proved as the main force to plasticize the cement particles. In fact, this force mainly depended on amount of S-PCE [62,63]. It was generally believed that a greater amount of S-PCE could provide higher dispersion capacity. However, if PEO were wrapped or covered by hydration products or precipitates, PEO would not be stretched into the solution to efficiently provide the steric hindrance, which meant that the wrapped or covered PCE would be invalided [64,65,66]. Based on this, the effect of phosphate on formation of S-PCE, which would obviously affect the water/solid interface performance at the very beginning, should be associated with the dispersion of PCE-phosphate system.

**Figure 15:**
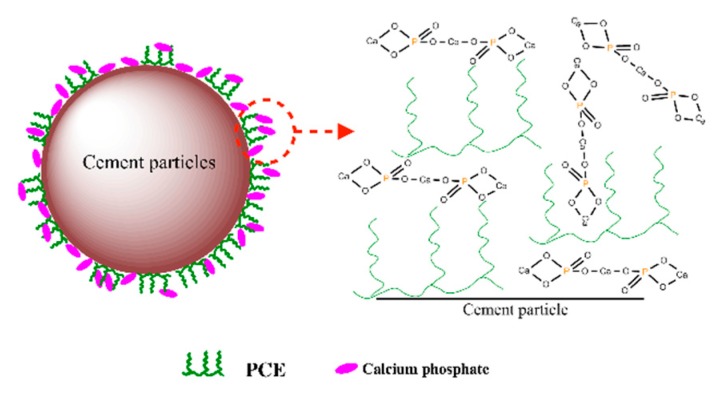
Dispersion model of PCE system with TSP.

In the PCE-TSP system, when the cement was mixed with PCE system, Ca^2+^ was released into the liquid phase and PCE quickly adsorbed onto surface of cement grains; simultaneously, TSP was precipitated with Ca^2+^ in the immediate vicinity of cement grains, which could alter the surface performance of these grains, thereby perturbing the adsorption of PCE. A more interesting phenomenon was that TSP would most likely be precipitated on the surface of S-PCE to cover and inactivate the S-PCE, as shown in Figure 15. In that case, PEO might be squeezed and it could not work normally, which meant that the covered S-PCE would be invalided and could not provide dispersion [64,67]. In fact, similarity to the PCE covered by hydration products, the covered S-PCE was deemed as the ineffective adsorption. As a consequence, the precipitation of calcium-based phosphate to cover and invalidate the S-PCE was responsible for the decrease in dispersion capacity of the PCE-TSP system.

**Figure 16:**
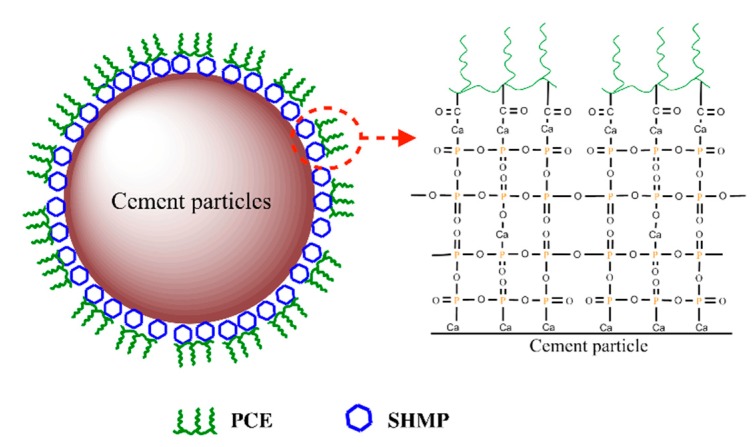
Dispersion model of PCE system with SHMP.

In the PCE-SHMP system, SHMP was combined with Ca^2+^ and Ca-O structure on the surface of cement particles. As the competitive adsorption would take place between PCE and SHMP, the adsorption points where PCE had adsorbed could not be adsorbed by SHMP, while PCE could not adsorb onto the point where it had been preferentially adsorbed by SHMP. However, the adsorption ability of SHMP was much stronger than that of PCE, which was proved before and, therefore, the vast majority of SHMP would preferentially adsorb to form the SHMP layer. PCE in solution continuously adsorbed onto the SHMP layer to form a S-PCE layer outside the SHMP layer over time. In other words, most PCE would adsorb onto the surface of the SHMP layer via Ca^2+^ in solution, as shown in Figure 16, to from the SHMP-Ca-PCE layer. Almost no PCE would be wrapped into the inside layer, which avoids the consumption of PCE caused by hydration products; this could enhance the dispersion efficiency of PCE system, in spite of much less adsorption amount of PCE. As a consequence, it was concluded that the increase in dispersion capacity of PCE-SHMP system was attributed to the enhancement of dispersion efficiency of PCE resulting from the formation of the multiple adsorption layer that was composed of several SHMP layers inside and a PCE layer outside.

## 4. Conclusions

TSP and SHMP both delayed the cement hydration, impeded the adsorbing process of PCE, and increased the thickness of adsorption layer, while an interesting result was found: TSP reduced the dispersion but SHMP was enhanced.

TSP was quickly precipitated with Ca^2+^ in the immediate vicinity of the cement particle, which could cover and invalidate the adsorbed PCE, thereby reducing the dispersion of PCE. SHMP was combined with Ca^2+^ and preferentially adsorbed onto the surface of cement particle, which could form “Inner-phosphate (multi-layers) + Outer-PCE (single layer)” structure, hence enhancing the dispersion.

## Figures and Tables

**Table 1:** Chemical composition of cement.

		LOI.	SiO_2_	Al_2_O_3_	Fe_2_O_3_	SO_3_	CaO	MgO	K_2_O	Na_2_O
Cement	wt%	3.72	22.79	7.03	3.14	3.76	55.05	2.90	0.57	0.14

**Table 2:** Basic performance of polycarboxylate superplasticizer (PCE).

Water Reducing Ratio (%)	Density (g/cm^3^)	pH Value	Solid Content (%)
32.1	1.076	7.0	40.0

**Table 3:** Solutions prepared in advance for adsorption measurement.

	PCE (g/L)	TSP (g/L)	SHMP (g/L)
Single-component system	1.00		
		0–2.0	
			0–2.0
Binary-component system	1.00	0–2.0	
	1.00		0–2.0

**Table 4:** Adsorption layer on the cement particles calculated from X-ray photoelectron spectrometer (XPS) data.

	Blank	TSP + PCE	SHMP + PCE	PCE
*hν*(eV)	1486.60	1486.60	1486.60	1486.60
E_b_ (eV)	101.98	101.97	101.97	101.97
Height Counts	1470.72	749.95	774.44	1013.12
FWHM (eV)	2.72	2.03	2.00	2.62
I_0_	4000.36			
I(b)		1522.40	1548.88	2654.37
I/I_0_		0.3805	0.3872	0.6635
Ek		1384.63	1384.63	1384.62
λ(Ek)		4.0932	4.0932	4.0932
Adsorption layer (b, nm)		3.95	3.88	1.68
